# The Origin and History of the N-Localizer for Stereotactic Neurosurgery

**DOI:** 10.7759/cureus.323

**Published:** 2015-09-14

**Authors:** Russell A Brown, James A Nelson

**Affiliations:** 1 Software Development, High Technology; 2 Radiology (Emeritus), University of Washington

**Keywords:** stereotactic neurosurgery, stereotactic radiosurgery, image guidance, image-guided, computed tomography, magnetic resonance imaging, positron emission tomography (pet), n-localizer, medical imaging, brain imaging

## Abstract

Nearly four decades after the invention of the N-localizer, its origin and history remain misunderstood. Some are unaware that a third-year medical student invented this technology. The following *conspectus* accurately chronicles the origin and early history of the N-localizer and corrects some misconceptions related to both.

## Introduction and background

**Figure 1:**
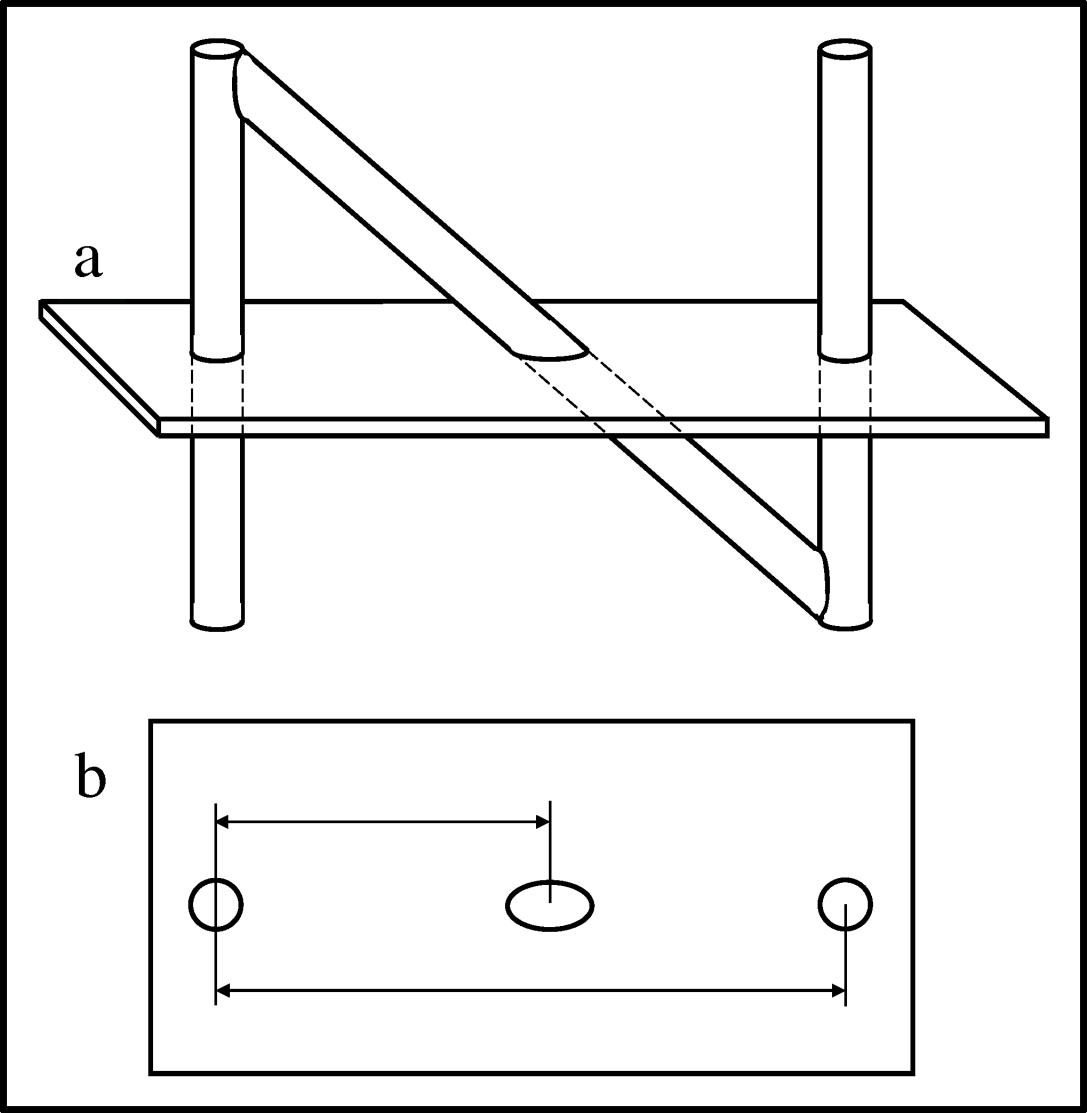
N-Localizer and Its Interaction with the Tomographic Section $\mathbf{(a)}$  Side view of the N-localizer. The tomographic section intersects the N-localizer at two vertical rods and one diagonal rod. $\mathbf{(b)}$ Tomographic image. The intersection of the tomographic section with the N-localizer produces two circles and one ellipse. The relative spacing between the centers of the ellipse and the two circles varies according to the height at which the tomographic section intersects the diagonal rod. Measuring this spacing permits calculation of the position of the tomographic section with respect to the N-localizer [2].

The N-localizer has become an important tool for image-guided stereotactic neurosurgery and radiosurgery. The N-localizer produces two circles and one ellipse in tomographic images that are obtained via computed tomography (CT), magnetic resonance imaging (MRI) or positron emission tomography (PET). The relative spacing between the ellipse and the two circles precisely determines the position of the tomographic section with respect to the N-localizer (Figure 1) [1-2].

**Figure 2:**
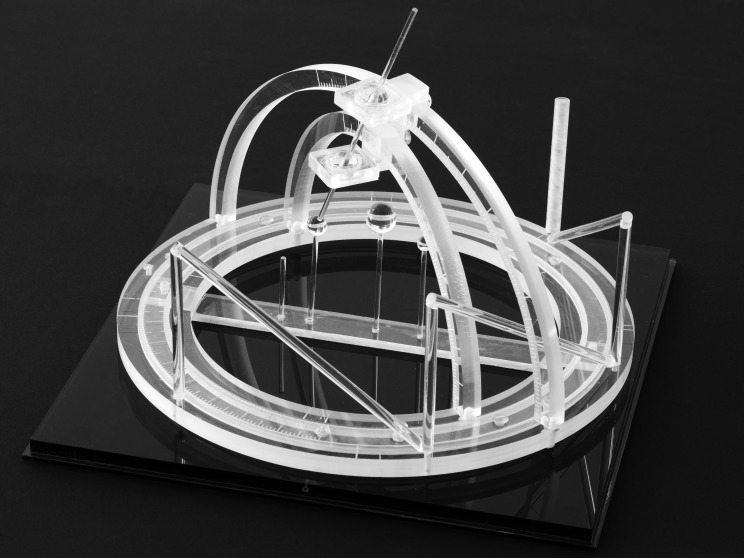
The First CT-Compatible Stereotactic Frame Brown designed and built this stereotactic frame in August 1978 in order to test the concept of the N-localizer [1]. Three N-localizers are attached to this frame and are merged end-to-end such that only seven rods are present. Because three points determine a unique plane in three-dimensional space, the locations of the centers of the three ellipses that are produced in a tomographic image by three N-localizers precisely determine the position of the tomographic section with respect to the stereotactic frame [2].

Russell A. Brown invented the N-localizer in May 1978 when he was a third-year medical student and during a research elective under the supervision of James A. Nelson at the University of Utah [3]. In August 1978, Brown designed and built the first CT-compatible stereotactic frame in order to test the concept of the N-localizer (Figure 2). This stereotactic frame was presented at a joint meeting of the Western Neurological Society and the American Academy of Neurological Surgery held in Los Angeles, California in October 1978 [1] and at the INSERM Symposium on Stereotactic Irradiations held in Paris, France in July 1979 [4].

Beginning in 1979, six different stereotactic frames incorporated the N-localizer: the Brown-Roberts-Wells (BRW) frame [5], the Pfizer frame that was used at the University of Pittsburgh [6], the Kelly-Goerss frame [7], the modified Reichert-Mundinger frame that was used at Duke University [8], the Leksell frame [9], and the Cosman-Roberts-Wells (CRW) frame [10]. Subsequently, the N-localizer achieved widespread use in image-guided stereotactic neurosurgery and radiosurgery [11-40]. The simplicity and accuracy of the N-localizer render it an important tool for modern neurosurgery [3].

## Review

During the 37 years since the invention of the N-localizer, some misconceptions have arisen concerning its origin and history in relation to subsequent developments in image-guided stereotactic surgery.

The first misconception is that the Pfizer frame, which incorporated the N-localizer, was constructed and initially used in 1978. Kondziolka and Lunsford of the University of Pittsburgh assert this misconception, together with their failure to discuss the relevant literature, in their claim [41], "At our center, the first CT compatible stereotactic head frame, in collaboration with industry, was constructed in 1978 and utilized in 13 patients [6,42]. *[...]* During this interval, the newly redesigned Leksell CT compatible stereotactic head frame [43] was used for dedicated brain biopsies under the direction of its inventor, Lars Leksell. Several groups were working on devices to allow accurate CT based stereotactic surgery [44].”

The above assertion presents an erroneous chronology. The Pfizer frame was neither the first CT-compatible stereotactic frame (Figure 2) nor was it constructed and initially used in 1978. Instead, it was constructed and initially used in 1979, as per Lunsford, *et al*., who recount, "In 1979, our first efforts in image-guided stereotactic surgery attempted to adapt an early-generation Leksell frame. The metallic artifacts precluded adequate computerized tomography (CT) imaging, and we subsequently developed a CT-compatible stereotactic device (Pfizer frame *[...]* ) [45,6] which was used in an initial series of 15 patients beginning in 1979" [26]. This statement is corroborated by Lunsford, Niranjan, Kassam, Khan, Amin and Kondziolka, who state, "During the interval of 1979 to 1980, 13 stereotactic procedures were performed in a diagnostic scanner at our hospital" [46]. These two statements confirm that the Pfizer frame was constructed and initially used in 1979, not in 1978.

The two above statements of Lunsford, *et al*. are corroborated by Perry, Rosenbaum, Lunsford, Swink and Zorub, who state that the Pfizer "stereotactic frame was made after attempts to modify the Leksell frame *[...]* proved difficult" [6]. Further corroboration is provided by a letter from Perry to Lunsford, Rosenbaum and Zorub [47] and a letter from Pfizer Medical Systems, Inc. to its patent attorney [48]. These letters verify that as of January 15, 1979, Perry, Rosenbaum, Lunsford and Zorub had not yet attempted any surgery using the modified early-generation Leksell frame. Hence, the Pfizer frame, which was constructed after efforts to adapt the early-generation Leksell frame had failed, was constructed no earlier than 1979.

The above assertion of Kondziolka and Lunsford disregards the fact that the CT-guidance technologies of the Leksell frame and the Pfizer frame were derivative. For both the Leksell and the Pfizer CT-compatible stereotactic frames, the inclusion of vertical and diagonal elements originated from Brown's prior invention and description of the N-localizer. This fact is established by the articles that introduced the Leksell [43] and Pfizer [6] frames. Both articles cited one [1] of Brown's original articles that had introduced the N-localizer one year earlier [1-2]. Although Lunsford (with and without Kondziolka) had previously cited [6,11,40,45-46,49] either of Brown’s original articles [1-2], these coauthors cited neither of his original articles in their above assertion [41]. Instead, they cited a later article by Roberts and Brown [44] that was published contemporaneously with the first articles from the University of Pittsburgh [6,42] and one year after Brown’s original articles had introduced the N-localizer [1-2].

The second misconception is that investigators from Pfizer and the University of Pittsburgh invented the N-localizer. This misconception is asserted by Lunsford, Niranjan, Kassam, Khan, Amin and Kondziolka, who claim [46], “During the subsequent years of training, the senior author had an opportunity to work with an innovative neuroradiologist, Arthur Rosenbaum, M.D., and an engineer, John Perry, Ph.D., who then headed the imaging division of Pfizer Medical Instruments. Together, we developed an image-guided stereotactic system using the now well-known N-localizer technology. This elegant solution was proposed by Perry et al. [6] and Rosenbaum et al. [42] independently and virtually simultaneously as publications from Brown [2] and Roberts and Brown [44] of Utah."

In the above assertion, the intended antecedent of “elegant solution” could be either “image-guided stereotactic system” or “N-localizer technology.” Perry, *et al*. did propose the Pfizer image-guided stereotactic system [6] several months after Brown, *et al*. had proposed the Brown-Roberts-Wells (BRW) image-guided stereotactic system [5]. However, the historical record shows that none of the above-mentioned individuals, with the exception of Brown, invented the N-localizer. Instead, Perry adopted the N-localizer after Brown had disclosed it to him. Documents that corroborate these facts have remained preserved in the archives of the U.S. Patent and Trademark Office for the past 30 years. The following discussion, which is based on those archives, recounts Perry's research related to image-guided stereotactic surgery and reveals the events that led to his adoption of the N-localizer.

**Figure 3:**
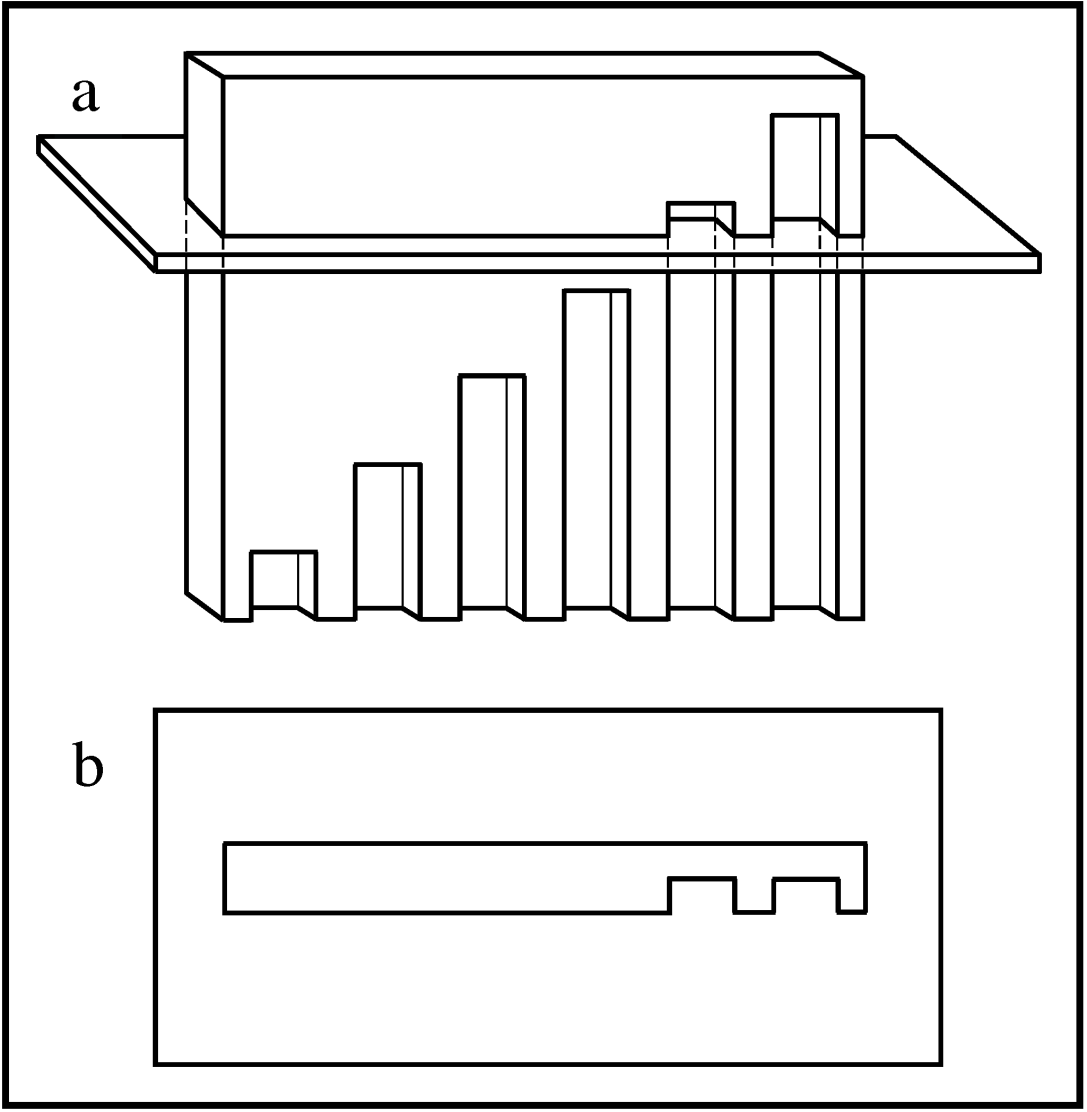
Slotted Plate and Its Interaction with the Tomographic Section $\mathbf{(a)}$  Side view of the slotted plate. The tomographic section intersects the plate into which are milled vertical slots of different lengths. The tops of the slots lie along a diagonal line. $\mathbf{(b)}$ Tomographic image. The intersection of the tomographic section with the slotted plate produces a variable number of notches. The number of notches depends on the height at which the tomographic section intersects the plate. Counting the number of notches permits estimation of the position of the tomographic section with respect to the slotted plate.

Prior to the invention of the N-localizer, several coauthors had reported a method for estimating the position of a tomographic section with respect to patient anatomy [50-51]. That method involved a plate into which were milled vertical slots whose tops lay along a diagonal line (Figure 3).

Documents from the archives of the U.S. Patent and Trademark Office indicate that as of January 15, 1979, Perry, Rosenbaum, Lunsford and Zorub had attached three slotted plates to a Leksell frame [47-48]. In principle, three slotted plates could enable the calculation of the position of a tomographic section with respect to a stereotactic frame, similar to the manner in which three N-localizers enable this calculation (Figure 2).

In practice, however, the slotted plate was susceptible to error as a result of the discrete or quantized nature of the slots. Perry observed that it was necessary to manually count carefully the numerous notches that were visible in the tomographic image because any miscount would give rise to errors in the subsequent calculation of the position of the tomographic section with respect to the stereotactic frame [47]. Moreover, the partial volume effect [52-53], which derives from the several-millimeter thickness of the tomographic section, impeded accurate counting of the notches because any slot that passed partially into but not entirely through the tomographic section would produce an only faintly visible notch. For these reasons, the slotted plate was vulnerable to human error and hence was unsuitable for clinical use. The N-localizer avoids these quantization problems and the attendant possibility of computational errors by virtue of the continuous nature of the N-localizer's rods.

Perry's earliest report of the slotted plate, and indeed the earliest record of his involvement with image-guided stereotactic surgery, was in his letter dated January 15, 1979, and addressed to his collaborators, Lunsford, Rosenbaum and Zorub at the University of Pittsburgh [47]. Perry's letter describes three slotted plates attached to a stereotactic frame and provides instructions for using computer software in conjunction with those slotted plates to calculate the position of a tomographic section with respect to that stereotactic frame. Well before the date of Perry's letter, Brown had already invented the N-localizer [54], built the first CT-compatible stereotactic frame [55], and presented his results to the Western Neurological Society and the American Academy of Neurological Surgery [1].

On January 25, 1979, Brown spoke by phone with one of Perry's coworkers at Pfizer Medical Systems, Inc. and learned that Perry's research involved image-guided stereotactic surgery [56]. The following day, another of Perry's coworkers at Pfizer Medical Systems, Inc. sent to its patent attorney a letter that included a photo of a Leksell frame to which three slotted plates were attached and a photo of a CT scan image of the Leksell frame and slotted plates [48].

A few days following his conversation with Perry's coworker, Brown spoke by phone with Perry and disclosed the N-localizer to him [57]. Prior to this discussion with Brown, Perry had been unaware of the concept of the N-localizer. Perry may have apprised Rosenbaum of some aspects of this discussion with Brown. Nelson affirms that, during a conversation with Rosenbaum concerning the N-localizer, Rosenbaum revealed his awareness of Brown's prior discussion with Perry [57].

Several months following his discussion with Perry, Brown was surprised to witness a talk wherein Perry presented the N-localizer without attributing its origin to Brown [57]. When Perry, *et al.* subsequently proposed the Pfizer image-guided stereotactic system [6], which comprised N-localizers instead of slotted plates, they cited one [1] of Brown's original articles that had introduced the N-localizer one year earlier [1-2]. Several months before Perry, *et al.* proposed the Pfizer image-guided stereotactic system, Brown, *et al.* had already proposed the Brown-Roberts-Wells (BRW) image-guided stereotactic system [5].

The efforts of Perry, *et al.* to adapt an early-generation Leksell frame for CT imaging by attaching three slotted plates to that frame were unsuccessful [6, 26]. Perry, *et al.* abandoned the slotted plate, adopted instead the N-localizer, and never published a description of the slotted plate attached to a stereotactic frame.

However, Perry himself described three slotted plates attached to a stereotactic frame in his application to the U.S. Patent and Trademark Office dated April 13, 1979. The resulting patent issued on July 27, 1982 [58], and was the first public disclosure of Perry's technique of attaching three slotted plates to a stereotactic frame. Prior to that first public disclosure, Perry had disclosed privately to Brown, *circa* January 1979, three slotted plates attached to a stereotactic frame. An entry in Brown's notebook recounts his phone conversation with Perry that occurred *circa* January 1979. That entry includes a drawing of the slotted plate and reports that “John Perry of Pfizer began working on a localizing system, according to him in the fall of 1978. This system, as I understand it, consisted of 3 plates having vertical grooves in them” [57]. Brown's drawing and report, which are dated October 14, 1979, prove his awareness of Perry’s slotted-plate technique three years prior to the first public disclosure of that technique and hence corroborate Brown's account of his phone conversation with Perry.

Perry's earliest description of the N-localizer was cursory and limited to only two sentences in his patent that devoted detailed explanations and five drawings to a thorough description of his slotted-plate technique [58]. When challenged by Brown via a Patent Interference proceeding before the U.S. Patent and Trademark Office, Perry failed to provide any evidence whatsoever of having invented the N-localizer. Consequently, Perry conceded “priority of invention” to Brown [59] and the U.S. Patent and Trademark Office awarded patent protection for the N-localizer to Brown [60]. The documents [1, 47-48, 54-56, 59] that the U.S. Patent and Trademark Office examined prior to awarding patent protection to Brown instead of Perry are a matter of public record. Those documents may be obtained from the U.S. Patent and Trademark Office by requesting a copy of the folder for Patent Interference No. 101267. In order to facilitate access to those documents, copies are included in the Appendices to this article.

## Conclusions

Brown invented the N-localizer and built the first CT-compatible stereotactic frame in 1978. The N-localizer has become an important tool for modern neurosurgery and has achieved widespread use in image-guided stereotactic neurosurgery and radiosurgery. Beginning in 1979, six different stereotactic frames incorporated the N-localizer. For each frame, the inclusion of the N-localizer was derivative and originated from Brown's prior research.

## Figures and Tables

**Figure 4:**
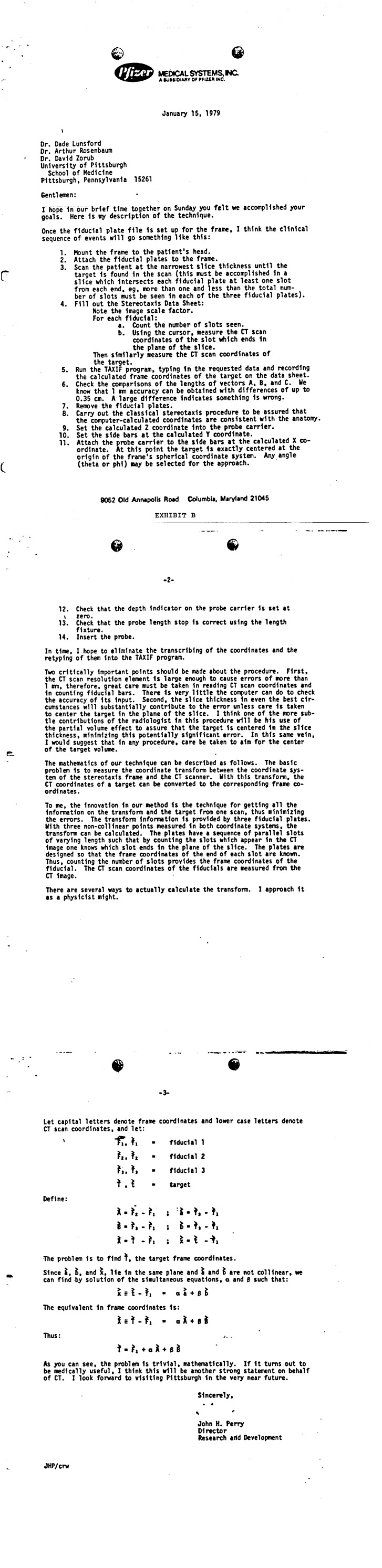
Appendix 1: John Perry Letter, pp. 1-3, January 15, 1979

**Figure 5:**
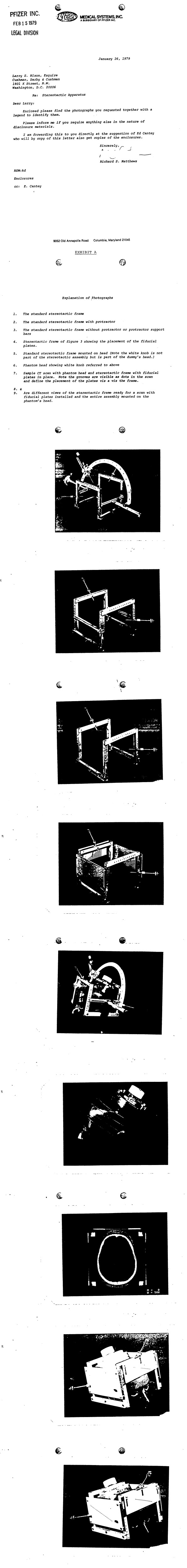
Appendix 2: Richard Matthews Letter, pp. 1-7, January 26, 1979

**Figure 6:**
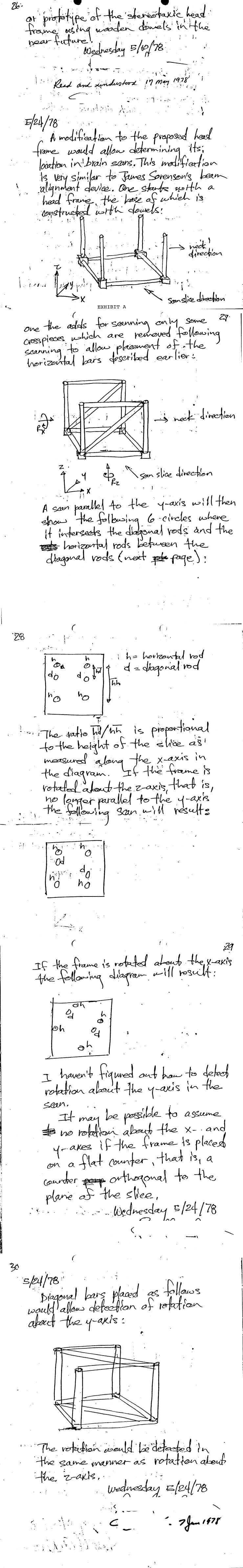
Appendix 3: Russell Brown Notebook 1, pp. 26-30, May 24, 1978

**Figure 7:**
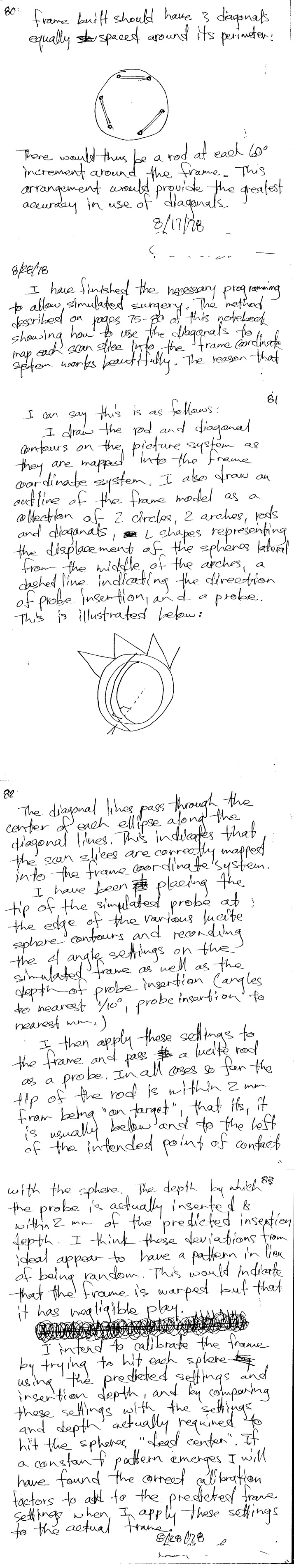
Appendix 4: Russell Brown Notebook 1, pp. 80-83, August 28, 1978

**Figure 8:**
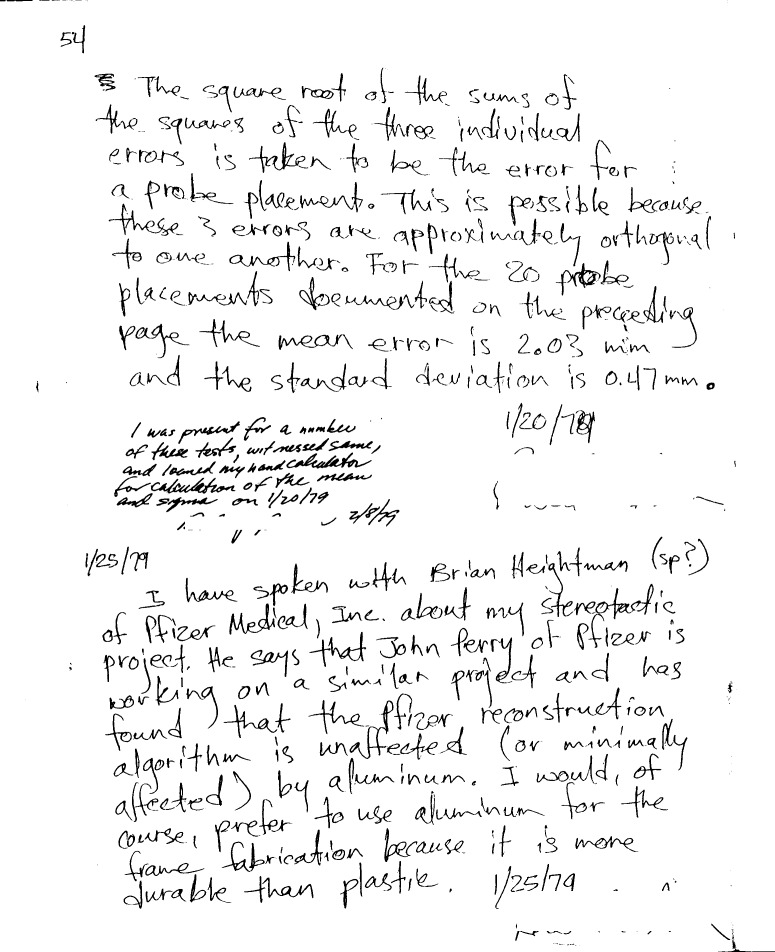
Appendix 5: Russell Brown Notebook 2, p. 54, January 25, 1979

**Figure 9:**
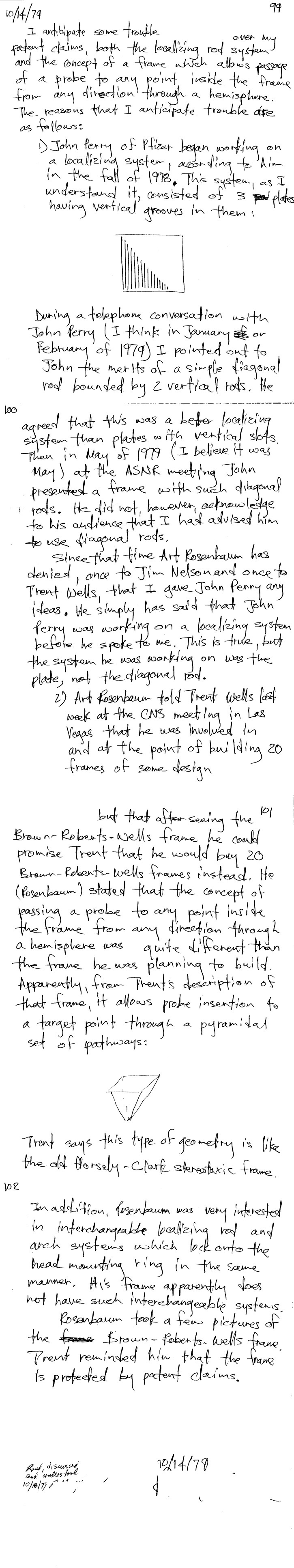
Appendix 6: Russell Brown Notebook 3, pp. 99-102, October 14, 1979

**Figure 10:**
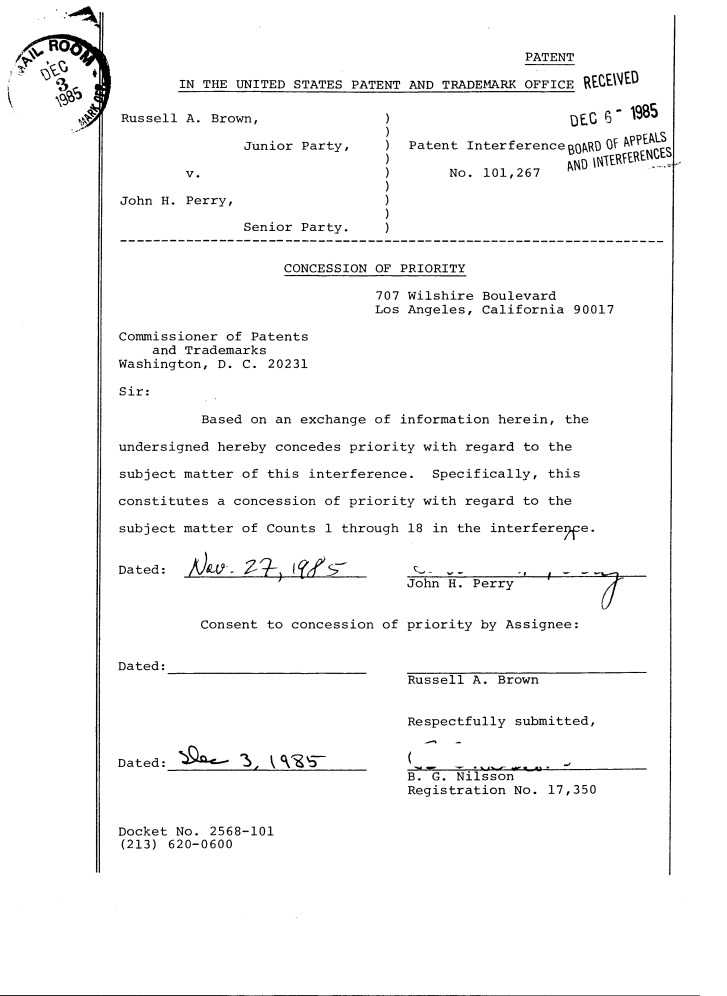
Appendix 7: John Perry Concession of Priority, November 27, 1985
